# Fungal diversity in deep-sea sediments from the Magellan seamounts as revealed by a metabarcoding approach targeting the ITS2 regions

**DOI:** 10.1080/21501203.2020.1799878

**Published:** 2020-08-02

**Authors:** Ye Luo, Xu Wei, Shuai Yang, Yuan-Hao Gao, Zhu-Hua Luo

**Affiliations:** a Key Laboratory of Marine Biogenetic Resources, Ministry of Natural Resources, Third Institute of Oceanography, Xiamen, PR China; b School of Marine Sciences, Nanjing University of Information Science & Technology, Nanjing, PR China; c Co-Innovation Center of Jiangsu Marine Bioindustry Technology, Jiangsu Ocean University, Lianyungang, PR China

**Keywords:** Deep-sea sediment, environmental factors, fungal diversity, high-throughput sequencing, seamount area

## Abstract

Recent reports have revealed diverse and abundant fungal communities in the deep-sea biosphere, while their composition, distribution, and variations in seamount zones are poorly understood. Using a metabarcoding approach targeting the ITS2 regions, we present the structure of the fungal community in 18 sediment samples from the Magellan seamount area of the northwest Pacific.

A total of 1,979 fungal OTUs was obtained, which were taxonomically assigned to seven phyla, 17 classes, 43 orders, 7 families, and 98 genera. The majority of these OTUs were affiliated to Basidiomycota (873 OTUs, 44.11% of total OTUs) and Ascomycota (486 OTUs, 24.56% of total OTUs), followed by other five minor phyla (Mortierellomycota, Chytridiomycota, Mucoromycota, Glomeromycota, and Monoblepharidomycota). Sordriomycetes is the most abundant class, followed by Eurotiomycetes, and Dothideomycetes. Five genera were common in most of the samples, including worldwide reported genera *Aspergillus, Cladosporium, Fusarium, Chaetomium*, and *Penicillium*. The environmental data we collected (sampling depth, sampling location latitude and longitude, organic carbon content, and organic nitrogen content in the sediment) had no significant influence on the composition and distribution of fungal communities. Our findings provide valuable information for understanding the distribution and potential ecological functions of fungi in the deep-sea sediments of the Magellan seamounts.

## Introduction

Deep-sea (> 1000 m) covers more than 65% of the Earth’s surface and fulfils a range of key ecosystem functions (Danovaro 2012). Although the deep-sea environment is characterised by the absence of sunlight irradiation, predominantly low temperature, and high hydrostatic pressure, the fungal community is diverse in this extreme environment where fungi are major components of microeukaryotes and play critical roles (Nagano and Nagahama 2012). Since the first report of deep-sea fungi isolated from the Atlantic Ocean at a depth of 4,450 m (Roth et al. 1964), an increasing number of fungal species was found in several deep-sea environments, e.g.: sediments from the Mariana Trench (Takami et al. 1997), the Yap Trench (Xu et al. 2019), the hydrothermal site of South Mid-Atlantic Ridge (Xu et al. 2017), calcareous sediments (Raghukumar and Raghukumar 1998), the Chagos Trench (RaghuKumar et al. 2004), the Central Indian Basin (Damare et al. 2006; Singh et al. 2010), and the deep-sea coral (Galkievicz et al. 2012). These studies clearly illustrate the increasing attention being paid to fungal abundance and diversity in deep-sea environments.

Microorganisms have also been found in the deep-sea area of the Pacific Ocean, such as the deep-sea volcano (Akerman et al. 2013), hydrothermal vent (Fortunato and Huber 2016), and water column (Li et al. 2019). The fungal community in deep-sea sediments from the different Pacific area has been reported before (Zhou et al. 2007; Burgaud et al. 2009, 2010; Nagano et al. 2010; Nagahama et al. 2011; Rédou et al. 2015; Zhang et al. 2015; Xu et al. 2014, 2016, 2018a, 2019). However, it is still insufficient when comparing Bacteria and Archaea communities (Wu et al. 2013; Luo et al. 2015; Zhang et al. 2015, 2018; Walsh et al. 2016; Bienhold et al. 2016; Peoples et al. 2019). Especially, there is a lack of information on fungal richness, diversity, and potential ecological roles in the Pacific seamount area.

Seamounts are undersea mountains that rise steeply from the sea bottom to below sea level, defined as having an elevation of more than 1000 m with a limited extent across the summit (Menard 1964). Seamounts in the world’s oceans are numerous, especially in the Pacific Ocean. The seamounts of the Pacific Ocean are old in history and undulating in terrain, giving birth to a unique deep-sea ecosystem (Geotimes, 1993; Kvile et al. 2014). Magellan seamounts chain locates in the western Pacific and consists of top flat seamounts (1500 m to 6000 m water depth) (Kellogg et al. 1987; Mel’nikov et al. 2009). The top flat seamount is characterised by a large flat roof and steep slope with a listric shape. The flat roofs are covered by Quaternary foraminifer sand and calcium ooze (Zhu et al. 2011). Up to now, previous studies have demonstrated that high productivity is a distinctive characteristic of seamounts because of a large number of organic matters providing sufficient matrix for growth of organisms (Genin and Boehlert 1985; Tseytlin 1985; Boehlert and Genin 2013). Previous studies have shown that seamounts are highly biologically diverse and have an abundance of biomes (Morato et al. 2010; Quattrini et al. 2015; Preez et al. 2016), discovering diverse microbial communities including bacteria (Ettoumi et al. 2010, 2013, 2016), archaea (Liao et al. 2011; Esther et al. 2015; Fortunato and Huber 2016) and fungi (Magnus et al. 2015). So far, however, there has been little concern about fungal diversity in sediments from the Magellan seamounts.

Several studies have shown that the potential drivers of the distribution of marine fungi could be specific environmental parameters, such as temperature, sample depth, and available nutrients (Booth and Kenkel 1986; Jones 2000; Jeffries et al. 2016; Tisthammer et al. 2016; Li et al. 2018). Globally, the distribution of marine fungi is related to temperature and salinity (Booth and Kenkel 1986). In marine sediments, environmental factors, especially sample depth, oxygen, and nitrate, have been found closely related to fungal community composition (Tisthammer et al. 2016). In the Arctic sediments, the diversity of fungi is mainly affected by salinity, organic carbon, silicate, and phosphate content (Zhang et al. 2015). In sediments of the margins of Peru, fungal communities and activities are associated with dissolved and total organic carbon and sulphide (Orsi et al. 2013). In deep-sea sediments of the Gulf of Mexico, the physical and chemical properties of sediments (water content, carbonate, nitrogen, and terrigenous content) and geographic location (region, latitude, longitude, and geographical distance) affect fungal community structure (Lluvia et al. 2019). These studies indicated that there is a relationship between fungal community structure and environmental factors. However, what is not yet understood is the relative importance of the various factors that function in different environments.

Our present knowledge of deep-sea fungal diversity is largely based on the identification of the fruiting body, culturing surveys and conventional sequencing of the internal transcribed spacer (ITS) of rRNA gene clones (RaghuKumar et al. 2004; Bass et al. 2007; Nagano et al. 2010; Singh et al. 2011, 2012; Xu et al. 2014, 2016; Zhang et al. 2016). High-throughput sequencing (HTS) of DNA amplification from marine environments is a powerful approach for screening fungal communities with better capacity for detecting rare species, the taxa that present only as vegetative mycelia and cannot be cultured (Zhang et al. 2016; Nagano et al. 2017; Wang et al. 2018; Xu et al. 2018b). A few of studies have been conducted to detect fungal assemblages present in bathypelagic and abyssopelagic zones and other specialised deep environments including hydrothermal systems, methane-dominated regions, and deep subsurface sediments (Bass et al. 2007; Lai et al. 2007; Takeshita et al. 2007; Jebaraj and Raghukumar 2009; Le Calvez et al. 2009; Nagano et al. 2010; Nagahama et al. 2011; Singh et al. 2011, 2012; Thaler et al. 2012; Xu et al. 2014, 2016, 2017).

To better understand the fungal community in the deep-sea sediment of the Magellan seamounts, the nuclear internal transcribed spacer 2 (ITS2) region was used as a barcode and Illumina MiSeq as sequencing platform. The results of this study will allow us to determine the diversity distribution and composition of the fungal communities, providing new details of fungal communities in the deep-sea seamounts. Besides, we also evaluated the influences of geographic location and physicochemical parameters on the distribution of fungal communities.

## Materials and methods

### Sampling

**Figure 1.**
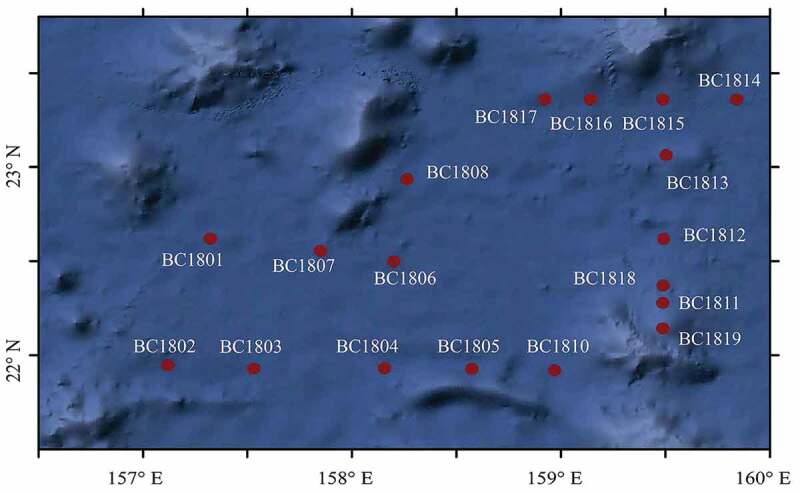

Using the Chinese scientific research vessel “Dayang No. 1”, sediment samples were collected from the northwest Pacific during the implementation of the Chinese Ocean 48 cruise from August 12 to 7 September 2018. The sampling location is in the centre of the Magellan Seamount chain (Zhao et al. 2010). Details of the collected samples were shown in Figure 1 and Table 1. Each sediment sample was divided into two fractions and stored at 4°C and −80°C, respectively, for subsequent separation and molecular analysis. Organic nitrogen and carbon were measured using CHNSO Elemental Analyser (Model FLASH2000, Thermo Scientific, USA) (Aoyagi et al. 2015).

**Table 1.** The information of the sample station.

Samples	Location		Depth (m)	Sediment type	Organic N%	Organic C%
	Longitude (°E)	Latitude (°N)				
BC1801	157.32	22.62	5437	Surface clay	0.06	0.34
BC1802	157.12	21.95	5412	Surface siliceous mud	0.10	0.42
BC1803	157.53	21.93	5426	Surface clay	0.09	0.40
BC1804	158.16	21.93	5474	Surface clay	0.09	0.39
BC1805	158.58	21.93	5496	Surface clay	0.09	0.37
BC1806	158.2	22.5	5442	Surface clay	0.09	0.47
BC1807	157.85	22.56	5396	Surface clay	0.08	0.45
BC1808	158.25	22.94	5269	Surface clay	0.08	0.38
BC1810	158.97	21.92	5255	Surface clay	0.10	0.71
BC1811	159.49	22.28	4132	Surface calcareous slime	0.07	0.25
BC1812	159.49	22.62	5087	Surface clay	0.08	0.34
BC1813	159.5	23.06	5270	Surface clay	0.08	0.32
BC1814	159.84	23.36	5488	Surface clay	0.09	0.52
BC1815	159.48	23.36	5417	Surface clay	0.08	0.38
BC1816	159.12	23.36	5240.38	Surface clay	0.09	0.40
BC1817	158.9	23.36	5421.31	Surface clay	0.09	0.44
BC1818	159.49	22.37	4508.03	Surface clay	0.08	0.41
BC1819	159.48	22.14	3530.98	Surface calcareous slime	0.09	0.39

### DNA isolation

The environmental genomic DNA of the sediment samples was extracted by FastDNA®Spin Kit for Soil (MP bio, Santa Ana, USA), according to the manufacturer’s instruction. The ITS region of the fungal ribosomal RNA gene was amplified by PCR in 50-μl reactions (95°C for 2 min, followed by 27 cycles at 98°C for 10 s, 62°C for 30 s, and 68°C for 30 s and a final extension at 68°C for 10 min) (Xu et al. 2019). The primer sequences were: ITS3- KYO2 F: GATGAAGAACGYAGYRAA; ITS4-R: TCCTCCGCTTATTGATATGC (Toju et al. 2012). The PCR amplified product was then recovered and quantified using the QIAquick PCR purification kit (Qiagen) and Qubit 3.0 (Thermo Scientific). Sequencing libraries were generated using NEB Next R Ultra TM DNA Library Prep Kit for Illumina (NEB, USA) and added index codes. The library quality was assessed by the QuantiFluorTM-ST Blue fluorescence quantitative system (Promega) and sequenced by paired-end (2 × 250 bp) Illumina HiSeq 2500 platform at Genedenovo Inc. Guangzhou, China. The raw reads were deposited into the NCBI Sequence Read Archive (SRA) database (Accession Number: SAMN14543412-SAMN14543447)

### Quality control and reads assembly

Raw sequencing data obtained included dirty reads containing adapters or low-quality bases which would affect sequence assembly and analysis. To get high-quality clean reads, raw reads were filtered according to the following rules: 1) removing reads containing more than 10% of unknown nucleotides (N); and/or 2) removing reads containing less than 80% of bases with quality (Q-value)>20 (Lu et al. 2014; Li et al. 2017; Xu et al. 2018b). Paired-end clean reads were merged as raw tags using FLASH (version 1.2.11) with a minimum overlap of 10 bp and mismatch error rates of 2%. Noisy sequences of raw tags were filtered by QIIME (version 1.9.1) (Caporaso et al. 2010) pipeline under specific filtering conditions (Bokulich et al. 2013) to obtain high-quality clean tags. Clean tags were searched against the reference database (http://drive5.com/uchime/uchime_download.html) to perform reference-based chimera checking using the UCHIME algorithm (Edgar et al. 2011). This analysis was performed on USEARCH (http://www.drive5.com/usearch/manual/uchime_algo.html) (Alloui et al. 2015). All chimeric tags were removed and effective tags were obtained for further analysis (Haas et al. 2011). The software MOTHUR (version 1.39.1) (Schloss et al. 2009) was used to remove redundant tags to get unique tags.

### Diversity analysis

The clean reads were clustered into operational taxonomic units (OTUs) of ≥97% similarity using the UPARSE (version 9.2.64) (Edgar 2013). The sequence with the highest abundance was selected as a representative sequence within each cluster. Venn analysis was performed in R to identify unique and common OTUs between-groups using Venn Diagram (version 1.6.17) and UpSet R (version 1.3.3) (Lex and Gehlenborg 2014; Lex et al. 2014). The representative sequences were classified into organisms by a naive Bayesian model using the RDP classifier (version 2.2) (Wang et al. 2007) based on the UNITE database (https://unite.ut.ee/, version 2016.11.20) (Kõljalg et al. 2005). Diversity indices including Chao1value, ACE value, Shannon index, and Simpson index were calculated in QIIME (Paul and Josephine 2010). Rarefaction curves were generated based on the Chao1 value, Shannon index, and Simpson index. KRONA (version 2.6) was then used to interactively visualise the species annotation results (Ondov et al. 2011).

The composition of microbial communities in different samples was studies based on beta diversity analysis. First, use the software Muscle (version 3.8.31) (Edgar 2004) to perform multiple sequence alignment based on the OTU sequences of all samples. Combined with abundance information of the OTU, the GUniFrac (version 1.0) package in the R language was used to calculate the Unweighted Unifrac and Weighted Unifrac distance between pairs of samples (Catherine and Knight 2005). We visualised patterns of variation in community composition using principle coordinates analysis (PCoA) by the cmdscale function with the stats package in R (version 3.2.1) (Cox and Cox 2008).

To further explore the differences in microbial community structure between samples, the Unweighted Pair-group Method with Arithmetic Means (UPGMA) was generated using the MOTHUR (version 1.39.1) (Schloss et al. 2009). To examine the relationship between microbial community structure and environmental factors, canonical correspondence analyses (CCA) were conducted using CANOCO software (Dang et al. 2010). The FUNGuild (version 1.0) database (https://github.com/UMNFuN/FUNGuild) was used to assign ecological functions (trophic modes) to all OTUs (Nguyen et al. 2016).

## Results

### Sequence analysis and OTU classification

**Figure 2.**
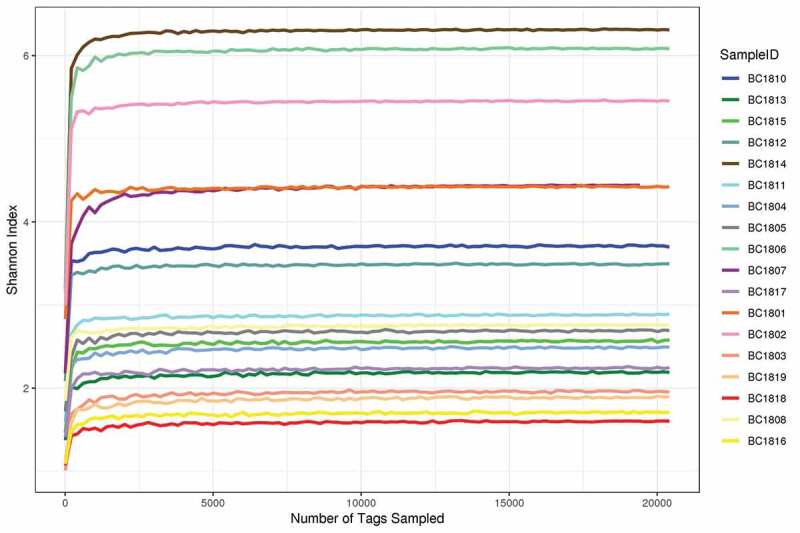

A total of 4548, 928 raw tags with 4536, 922 quality-filtered fungal ITS reads were obtained from the Illumina HiSeq 2500 platform sequencing. Previous studies have shown that removal of low-frequency sequences can reduce error rates and improve microbiota assessment (Tedersoo et al. 2010; ; Li et al. 2016). After filtration and denoising, a total of 1,979 OTUs at ≥ 97% similarity level was obtained from 18 sediment samples. The observed OTU richness and Shannon index were used for further analyses. The OTU richness of 18 samples differed from each other, ranging from 452–741 (Table 2). Shannon diversity ranged from 1.62–6.32 (Figure 2).

**Table 2.** Summary for pyrosequencing data and Alpha diversity index statistics from the 18 deep-sea sediment samples from the Magellan seamounts.

Sample name	Total Tags	Unique Tags	Taxon Tags	OTUs	ACE	Chao1	Simpson	Shannon	Coverage %
BC1801	333905	56051	333295	741	956.91	972.26	0.91	4.44	99.93
BC1802	442513	102539	441546	705	896.43	876.55	0.96	5.47	99.95
BC1803	173533	44072	172772	652	863.96	958.01	0.45	1.98	99.88
BC1804	173592	34598	172635	672	876.36	847.01	0.60	2.51	99.88
BC1805	303868	67530	302940	762	977.65	975.13	0.62	2.71	99.93
BC1806	431943	97404	427136	571	733.46	737.44	0.96	6.10	99.97
BC1807	20627	9074	19423	506	645.12	643.04	0.75	4.44	99.28
BC1808	237478	50021	236866	621	817.78	814.16	0.76	2.78	99.92
BC1810	227200	46678	226543	515	785.68	743.29	0.75	3.72	99.92
BC1811	262340	55193	261692	525	724.13	700.18	0.75	2.90	99.94
BC1812	314425	70658	313633	589	833.36	814.84	0.81	3.51	99.94
BC1813	197608	44125	196852	692	928.03	973.97	0.54	2.21	99.89
BC1814	338348	75770	335092	452	604.59	539.00	0.98	6.32	99.97
BC1815	294145	58515	293498	717	921.94	954.25	0.58	2.59	99.93
BC1816	202164	42840	201654	661	901.62	935.38	0.35	1.73	99.89
BC1817	197918	39058	197306	668	930.91	982.22	0.49	2.26	99.88
BC1818	213231	42427	212657	709	977.53	1015.96	0.44	1.62	99.89
BC1819	172084	38251	171392	738	963.21	1000.11	0.39	1.91	99.87

**Figure 3.**
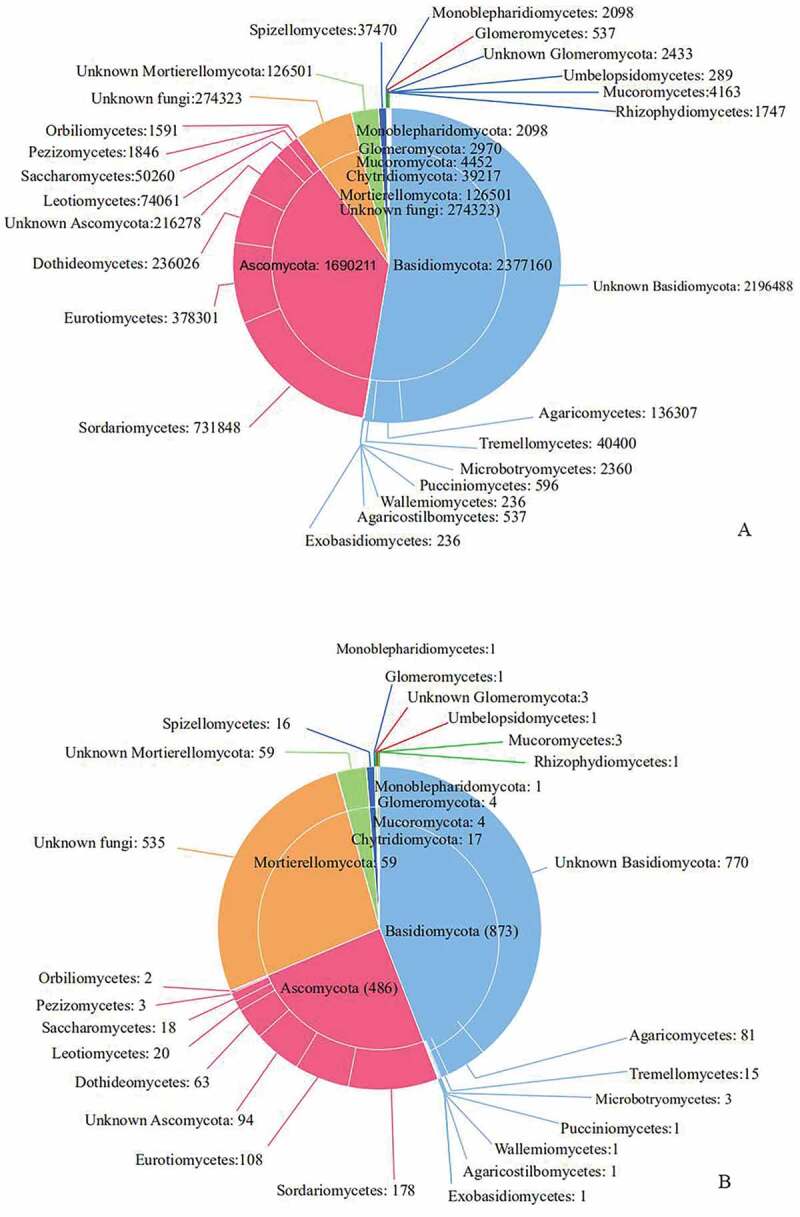

The taxonomical assigned OTUs belonged to seven phyla, 20 classes, 46 orders, 88 families, and 106 genera. Of the 1,979 fungal OTUs with 4536, 922 sequences, 873 (44.11%) were affiliated with Basidiomycota, followed by 486 (37.42%) with Ascomycota, 59 (2.98%) with Mortierellomycota, 17 (0.86%) with Chytridiomycota, 4 (0.20%) with Mucoromycota, 4 with Glomeromycota (0.20%), 1 (0.05%) with Monoblepharidomycota and 535 (27.03%) with unidentified fungi (Figure 3).

The sequences of Basidiomycota can be referenced in seven known classes, of which Agaricomycetes are the most abundant (136, 307 reads) and moderately diverse (81 OTUs), followed by Tremellomycetes (40, 400 reads and 15 OTUs), Microbotryomycetes (2, 360 and 3 OTUs), Pucciniomycetes (596 reads and 1 OTU), Agaricostilbomycetes (537 reads and 1 OTU), Exobasidiomycetes (236 reads and 1 OTUs) and Wallemiomycetes (236 reads and 1 OTU) (Figure 3).

Sordariomycetes (731,848 reads and 178 OTUs) in Ascomycota was the most abundant classes, followed by Eurotiomycetes (378,301 reads and 108 OTUs), Dothideomycetes (236, 026 reads and 63 OTUs), and Leotiomycetes (74, 061 reads and 20 OTUs), Saccharomycetes (50, 260 reads and 18 OTUs), Pezizomycetes (1, 846 reads and 3 OTUs), Orbiliomycetes (1, 591 reads and 2 OTUs) (Figure 3).

The 59 OTUs (1, 26501 reads) belonging to Mortierellomycota have no more detailed classification information. Spizellomycetes (37470 reads and 16 OTUs) is the most abundant in Chytridiomycota, and the other 1747 reads and 1 OTUs in Chytridiomycota were assigned to Rhizophydiomycetes. In Glomeromycota, only Glomeromycetes (537 reads 1 OTUs) and 3 OTUs (2, 433 reads) were identified. Mucoromycota includes Mucoromycetes (4, 163 reads, and 3 OTUs), Umbelopsidomycetes (289 reads, and 1 OTU). Only Monoblepharidiomycetes (2, 098 reads, and 1 OTU) can be recognised in Monoblepharidomycota (Figure 3).

**Figure 4.**
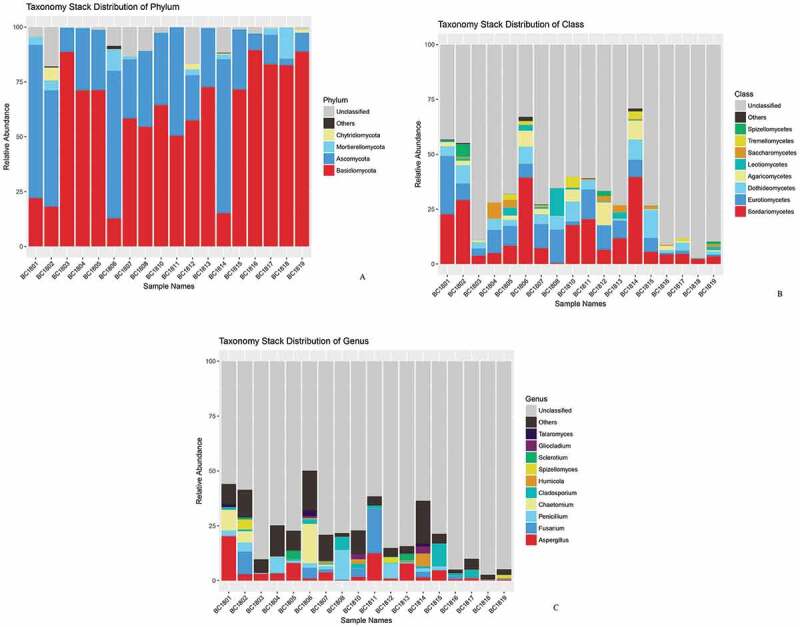

At the genus level, 106 fungal genera were identified, and 5 genera had great differences in sample richness, namely *Aspergillus, Cladosporium, Fusarium, Chaetomium*, and *Penicillium*, which accounted for 4.44%, 1.94%, 3.16%, 2.92% and 2.56% of the total sequences, respectively (Figure 4c). *Aspergillus, Cladosporium, Fusarium*, and *Penicillium* were detected in all samples.

### Fungal community composition in different samples

The relative abundance of fungal gates, classes, and genera were found to be different in different samples. In samples BC1801, BC1802, BC1806, and BC1814, Ascomycota accounted for more than 50% of the total sequences. In samples BC1804, BC1805, BC1813, BC1807, BC1808, BC1810, BC1811and BC1812, Basidiomycota accounted for more than 50% less than 75% of the total sequences. In the samples BC1803, BC1816, BC1817, BC1818, and BC1819, Basidiomycota accounted for more than 75% of the total sequences (Figure 4a).

The relative abundance of fungal classes and genera in the different samples were found to be different. Although diverse classes were recognised from samples, the Sordariomycetes, Eurotiomycete, and Dothideomycetes were the most abundant classes in the samples (Figure 4b). However, there are some fungal classes more abundant in some samples. To illustrate, Spizellomycetes was abundant in sample BC1802; Saccharomycetes was plentiful in sample BC1804; Leotiomycetes was abundant in sample BC1808; Agaricomycetes was ample in sample BC1812 (Figure 4b).

The most abundant genera recovered include *Aspergillus, Fusarium, Chaetomium, Penicillium, Cladosporium, Spizellomyces, Humicola, Sclerotium, Talaromyces, Gliocladium*. Among them, *Aspergillus, Cladosporium, Fusarium, Chaetomium, Penicillium* showed higher relative abundance in most sediment samples. Meanwhile, some fungal genera (eg, Humicola, Sclerotium, and Gliocladium) are plentiful in partial samples (Figure 4c).

**Figure 5.**
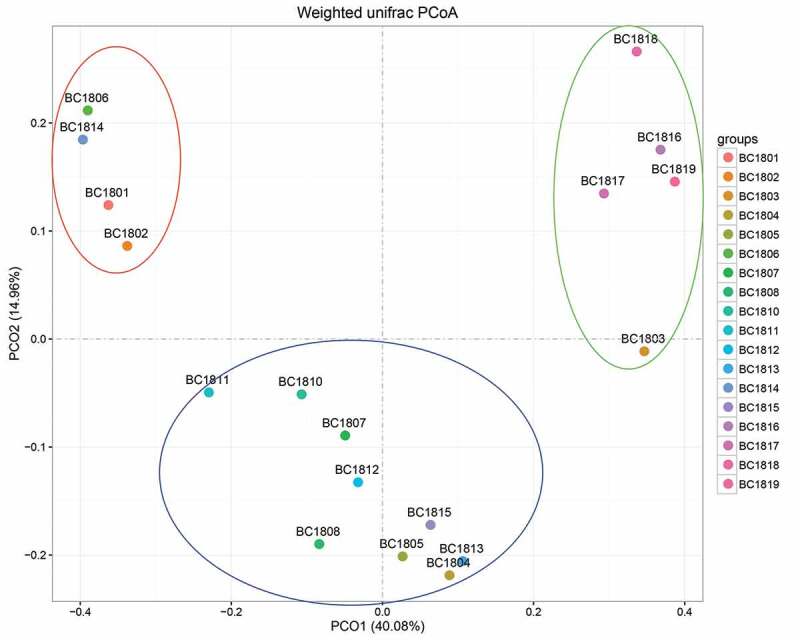

**Figure 6.**
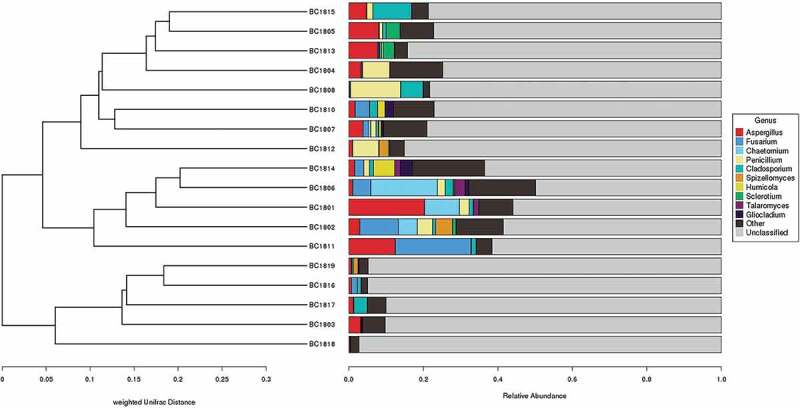

Principal coordinates analysis (PCoA) (Figure 5) based on the distribution of fungal OTUs exhibited a clear separation of fungal community structure between the 18 sediment samples, with the first principal component representing 29% of the total variation. Cluster analysis based on the 10 most abundant fungal genera using weighted unifrac distance analysis (UPGMA) (Figure 6) also provided a similar result with the fungal community structure between the 18 sediments. This difference in fungal communities between different sediment samples may be related to the differences between sediments, such as the spatial differences of sampling stations.

**Figure 7.**
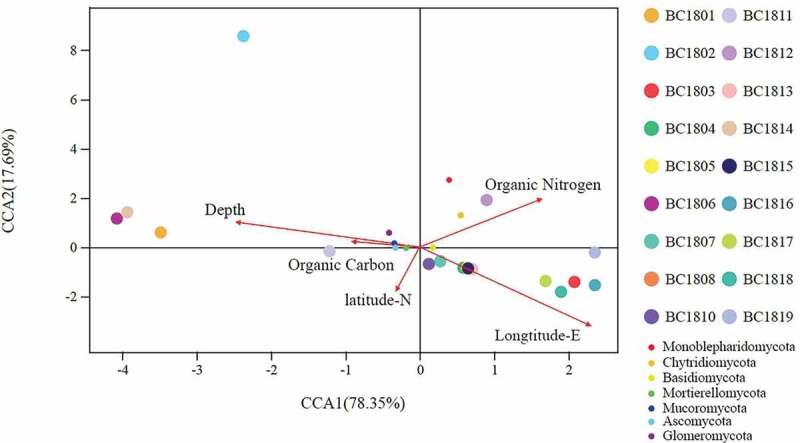

A further CCA analysis was performed to examine the relationship between microbial community structure and environmental factors (Figure 7). Among all environmental factors examined, organic Nitrogen (r^2^ = 0.1826, P = 0.205), organic Carbon (r^2^ = 0.0146, P = 0.859), depth (r^2^ = 0.0933, P = 0.401), Longitude-E (r^2^ = 0.1412, P = 0.288), Latitude-N (r^2^ = 0.0655, P = 0.616), no significant correlations were found between these environmental factors and fungi community structure (in all cases, P > 0.05). The present results show that among the analysed environmental factors, none of them has the significant effect on the composition and structure of the microbial communities.

**Figure 8.**
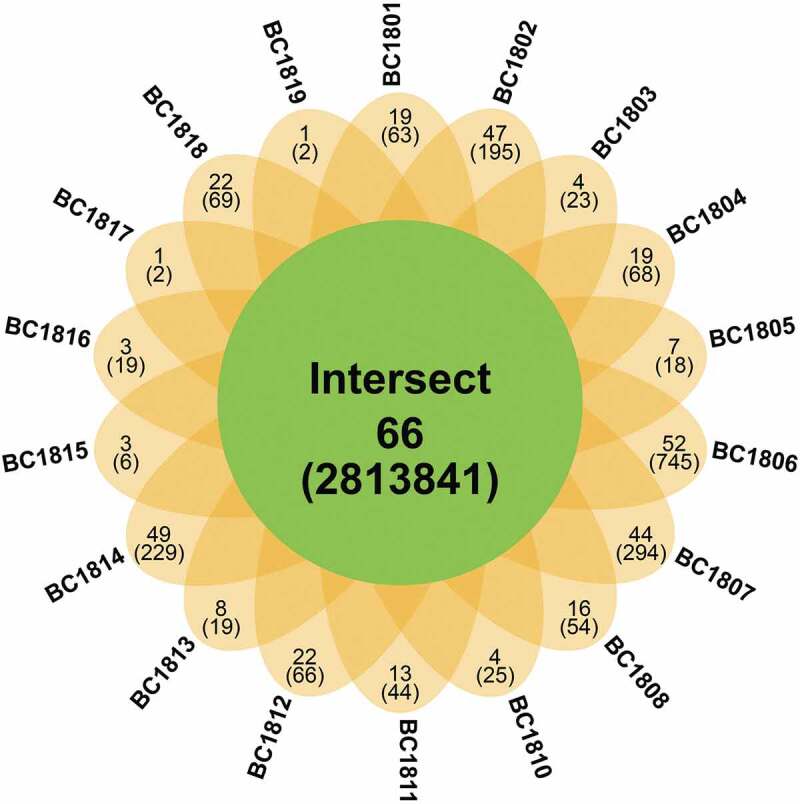

The core fungal taxa were referred to the OTUs shared by all samples, of which symbolise by the overlapping areas in the Venn diagram analysis. 66 fungal (281,384 reads) were shared by all 18 sediment samples and recognised as the core taxonomic group (Figure 8), which accounts for 3.33% of all fungal OTUs and 62.02% of total sequences. Some of these OTUs showed a high relative abundance in deep-sea sediments. For instance, OTU2 assigned to *Fusarium* and OTU6 assigned to *Aspergillus* account for 2.84% and 1.51% of the total fungal sequence, respectively. The samples BC1817 and BC1819 contained the lowest number of OTUs, while BC1806, BC1807, and BC1814 contained the highest number of OTUs. Besides, the sample-specific OTUs for each site ranges from 1 to 52. Most unique fungal OTUs were rare, less than 0.34% of total fungal sequences. The coexistence of 66 OTUs only accounts for 3.33% of the total 1979 OTUs. The core taxonomic group indicated the similarity of fungal communities among the 18 samples.

**Figure 9.**
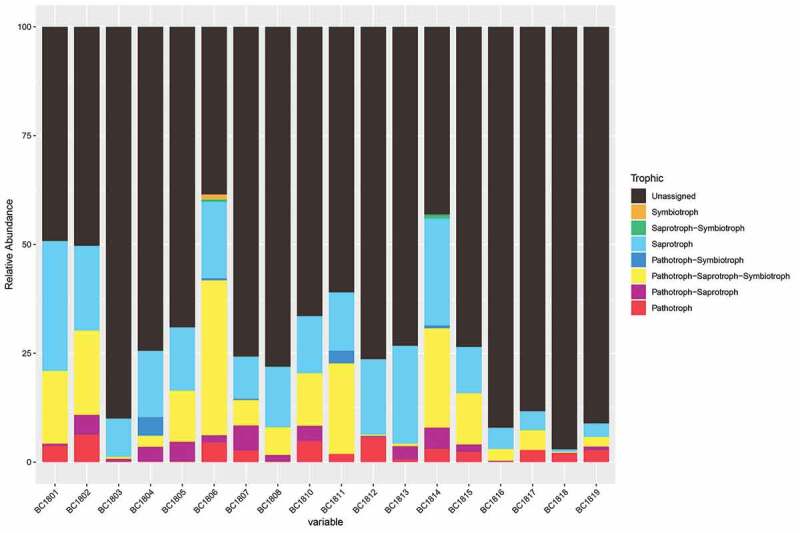

### FunGuild analysis

Based on the information of OTU taxonomic assignment and relative published articles, FUNGuild was used to predict the putative living strategies of fungi. Fungi recovered in this study can be characterised as Pathotroph-Saprotroph-Symbiotroph, Pathotroph-Saprotroph, Saprotroph-Symbiotroph, Pathotroph-Symbiotroph, Pathotroph, Saprotroph, Symbiotroph (Figure 9). Among them, Saprotroph was the most common life strategy.

## Discussion

Ascomycota and Basidiomycota have been recovered from other deep-sea habitats frequently (Nagano and Nagahama 2012; Xu et al. 2014, 2016, 2017, 2018b; Rédou et al. 2015; Nagano et al. 2017). High levels of Basidiomycota found in this study were different from most previous studies, of which described Ascomycota as the dominant phylum in the fungal community in other deep-sea habitats (Nagano and Nagahama 2012; Xu et al. 2014, 2016, 2017, 2018b; Rédou et al. 2015; Zhang et al. 2016; Nagano et al. 2017), indicating that the composition of fungal communities in this area may be unique. Similarly, Xu et al. (2019) presented high levels of Basidiomycota in several deep-sea sediment samples from Yap Trench by using high throughput sequencing. Ascomycota has been reported dominating the fungal community in deep-sea sediments worldwide, for instances: in the Southwest India Ridge of the Indian Ocean (Xu et al. 2018b), in the Okinawa Trough (Zhang et al. 2016) and the Sao Paulo Plateau of the Atlantic Ocean (Nagano et al. 2017). This deviance is probably due to the differences in the source of samples. Rämä et al. (2017) found that the dominant fungal phyla may be substratum-specific in the marine environment, Chytridiomycota, and Basidiomycota prevails in sea ice and seawater and Ascomycota overabundant on driftwood and sediments.

Analyses of environmental samples by molecular techniques recovered unknown clades from diverse marine ecosystems, especially in the deep-sea environment (Le Calvez et al. 2009; Nagano and Nagahama 2012; Xu et al. 2018b, 2019). Some phyla discovered in the deep-sea environment formerly were also recovered by a metabarcoding method in this study: Mortierellomycota (Xu et al. 2019), Chytridiomycota (Le Calvez et al. 2009; Nagano et al. 2010; Nagahama et al. 2011; Singh et al. 2011; Zhang et al. 2014, 2016; Xu et al. 2014, 2019), Mucoromycota (Xu et al. 2019), Glomeromycota (Le Calvez et al. 2009; Nagano et al. 2010; Nagahama et al. 2011). Monoblepharidomycota has been recovered from marine surface sediments previously but was firstly recovered from the deep-sea environment (Guo et al. 2015). Consistently with the previous study mentioned above, members of these phyla represented only small proportions of the sedimentary fungal communities. Nevertheless, Chytrids was found to dominate Arctic marine fungal communities and might change primary production patterns rapidly with increased light penetration through the Arctic Ocean (Hassett and Gradinger 2016). In marine habitats, Chytridiomycota, Mortierellomycota, and Mucoromycota have been characterised as decomposers of pollens and leaves (Phuphumirat et al. 2016) or pathogens of marine algae and animals (Scholz et al. 2016; Wang et al. 2018). These discoveries suggest that these fungi played a wide array of ecological roles potentially in the marine environment.

In this study, the most abundant class was the Sordariomycetes of Ascomycota, which is abundant in the marine environment. Previous studies have found that many obligate marine mycelium fungi belong to Sordariomycetes (Raghukumar 2017). Most classes recognised in this study were widely distributed in the deep-sea environment, as they were recovered from other regions of the deep-sea environment by molecular approach previously: Tremellomycetes, Microbotryomycetes, Agaricostilbomycetes, Exobasidiomycetes, Wallemiomycetes, Sordariomycetes, Eurotiomycetes, Dothideomycetes, and Leotiomycetes, Saccharomycetes, Pezizomycetes, Orbiliomycetes, Umbelopsidomycetes, Spizellomycetes, Pucciniomycetes and Glomeromycetes (Bass etal. 2007; Nagahama etal. 2011; Zhang etal. 2016; Nagano etal. 2017; Xu etal. 2018a, 2019). By ametabarcoding approach, we recovered Mucoromycetes, Rhizophydiomycetes, and Monoblepharidiomycetes in the deep-sea environments for the first time, updated the ecological distribution of these fungi. Some classes have been discovered from other environments, may be adapted to wide range habitats, such as Pucciniomycetes found from (Gao etal. 2010), Mucoromycetes found in the White Sea Sediments (Khusnullina etal. 2018), Monoblepharidiomycetes recovered from freshwater phytoplankton and lake samples (Ishida etal. 2015), Rhizophydiomycetes recovered in freshwater sites and high alpine exposed soils (Powell and Letcher 2014; Tedersoo et al. 2018).

*Aspergillus, Cladosporium, Fusarium, Penicillium* were detected in all deep-sea sediment samples. These genera have been widely recovered in deep-sea sediments around the world and were considered to be ubiquitous in the deep-sea environment (Nagano et al. 2010; Singh et al. 2012; Zhang et al. 2014; Rédou et al. 2015; Nagahama et al. 2011; Vargas-Gastelum et al. 2019; Xu et al. 2017, 2018a, 2018b, 2019). *Penicillium* and *Aspergillus* were amongst the most common genera in deep-sea ecosystems (Burgaud et al. 2009; Singh et al. 2010; Nagano and Nagahama 2012; Zhang et al. 2013; Xu et al. 2017, 2018b), as well as the most widely terrestrial forms of fungi in the sea and proved to be active in the marine environment owing to their physiological versatility (Raghukumar 2017).

Furthermore, the Venn diagram analysis revealed that the unique OTUs existed in every sample, reflecting the divergence among all the stations. These unique OTUs in each station may represent a rapidly changing community that is associated with the unique physicochemical properties of that location. While the common OTUs represent a more stable fungal community well adapted to habitat dynamics (Vargas-Gastelum et al. 2019).

Our study revealed a diverse fungal group in the deep-sea sediments of the Magellan Seamount by a metabarcoding approach, supplementing the fungal diversity information in this area. Compared with previous studies on fungal diversity of deep-sea sediments from other sites of the Pacific Ocean by using culture-dependent methods (Burgaud et al. 2009; Rédou et al. 2015; Xu et al. 2018a), clone libraries (Zhou et al. 2007; Nagano et al. 2010; Xu et al. 2014, 2016), studies by high throughput sequencing (Rédou et al. 2014; Zhang et al. 2015; Xu et al. 2019) notably extended our knowledge of the marine mycobiota in this area. Combining different methods for research could help us to understand the fungal diversity in the deep-sea environment more comprehensively.

However, 27.03% of the fungal OTUs could not be assigned to any fungal phyla based on the available databases. The high percentage of unidentified fungi was also detected in other deep-sea sediments, suggesting that there are largely unknown fungal taxa inhabiting in the deep-sea sediments, which probably includes indigenous fungi and species of potential biotechnological importance (Zhang et al. 2015; Barone et al. 2018; Vargas-Gastelum et al. 2019). This discovery of unidentified fungi may result from the insufficient coverage of ITS sequences in databases (Khomich et al. 2017). Currently, molecular studies have revealed a high diversity of ascomycetes and basidiomycetes in deep-sea with many novel lineages (Nagahama and Nagano 2012), while the low detection of taxonomic groups other than Ascomycota and Basidiomycota possibly results from the same reason (Tedersoo et al. 2015). The universal sequencing method targeting ITS2 regions are more likely to amplify ITS regions from Ascomycota and Basidiomycota, instead of other fungal groups (Op De Beeck et al. 2014; Amend et al. 2019). This limitation could mask the presence of other fungal groups. The use of multiple or group-specific primers might solve this obstacle (Singh et al. 2012a). Furthermore, a concordance between the rDNA and mRNA taxonomic diversity should be applied to estimate whether the fungi detected from the deep-sea environment by the sequencing approach are metabolically active.

Surprisingly, the canonical correspondence analysis revealed that all the environmental factors considered in this study (including organic Carbon, organic Nitrate, depth, longitude, and latitude) are not significantly related to fungal community composition. This result is different from other studies which showed that depth (Roth et al. 1964; Gong et al. 2015; Zhang et al. 2015), carbon content (Orsi et al. 2013; Zhang et al. 2015; Vargas-Gastelum et al. 2019) and sampling station latitude (Lluvia et al. 2019) were correlated with microbial community structure. These differences might result from the difference in sample source or insufficient environmental data. However, the organic nitrate, depth, longitude had more obvious influence on fungal community composition relatively. Global analysis of marine fungal community structure from water columns and sediments revealed that environmental factors, especially sample depth, oxygen, and nitrate are closely related to the fungal community composition (Tisthammer et al. 2016). This consistency suggested that the nitrate component of the sediments and sample depth are crucial factors to the community structure of marine fungal. Therefore, the distribution of marine fungi may depend on their interaction with a variety of environmental factors, which needs more environmental factors to do further analysis in subsequent studies.

Based on FUNGuild database analysis, saprotroph tends to be the most abundant life strategy. The higher abundance of saprotrophs in the deep-sea environment may be due to their key roles in decomposition processes. Saprotrophs are essential for nutrient turnover and sediment C storage. The saprotroph fungi in the deep-sea environment probably contributed to the maintenance of the sediment structure and nutrient cycling as to their great capability at producing extracellular enzymes in soil (Treseder and Lennon 2015). Agaricomycetes and Eurotiomycetes assigned as saprophytic fungi, which had been the most abundant and common classes in this study. (Cannon and Kirk 2007; Sterkenburg et al. 2015). *Aspergillus* and *Penicillium* are the major species of fungi in this study, also assigned as saprotrophs (Baldrian 2010). Some members of Dothideomycetes and Sordariomycetes had been discovered as plant pathogenic fungi, which contains *Fusarium, Chaetomium*, and *Cladosporium* (; Tedersoo et al. 2014; Lawrey and Diederich 2018). Another common life strategy is Pathotroph. Pathogenic fungi might accelerate the leaching out of Dissolved Organic Matter (DOM) from the host, which is then available to other microorganisms for their growth in the marine environment (Raghukumar 2017). Fungal functional groups may have important implications for the functions of various fungi which may reflect their function in the deep-sea environment.

In summary, our study characterised the fungal communities in deep-sea sediments of the Magellan Seamounts. We also explored the reasons for the divergence of the fungal community composition between different samples, which need to be further studied and anatomised in combination with more environmental factors. Nevertheless, our findings provide valuable information for understanding the distribution and potential ecological effects of fungi in the deep sea of the Magellan seamount area.
